# Toxoplasmosis Preventive Behavior and Related Knowledge among Saudi Pregnant Women: An Exploratory Study

**DOI:** 10.5539/gjhs.v5n5p131

**Published:** 2013-06-21

**Authors:** Tarek Tawfik Amin, Mohamed Nabil Al Ali, Ahmed Abdulmohsen Alrashid, Amena Ahmed Al-Agnam, Amina Abdullah Al Sultan

**Affiliations:** 1 Faculty of Medicine, Cairo University, Cairo, Egypt; 2 College of Medicine, King Faisal University, Al-Ahsa, Saudi Arabia; 3 Local Health Directorate, Primary Health Section, Ministry of Health, Al Hassa, Saudi Arabia

**Keywords:** toxoplasmosis, risk factors, preventive behavior, knowledge, pregnant women, Saudi Arabia

## Abstract

**Introduction:**

Many cases of congenital toxoplasmosis can be prevented provided that pregnant women following hygienic measures to avert risk of infection and to reduce severity of the condition if primary prevention failed.

**Objectives:**

This descriptive exploratory study aimed to assess the risk behavior and knowledge related to toxoplasmoisis among Saudi pregnant women attending primary health care centers (PHCs) in Al Hassa, Saudi Arabia and to determine socio-demographic characteristics related to risk behavior and knowledge.

**Methods:**

All Saudi pregnant women attending antenatal care at randomly selected six urban and four rural PHCs were approached. Those agreed to participate were interviewed using a pre-tested structured questionnaire collecting data regarding socio-demographic, obstetric history, toxoplasmosis risk behaviors and related knowledge.

**Results:**

Of the included pregnant women, 234 (26.8%) have fulfilled the criteria for toxoplasmosis preventive behavior recommended by Centers for Disease Prevention and Control to prevent congenital toxoplasmosis, while 48.9% reported at least one risk behavior and 24.3% reported ≥ two risk behaviors. Logistic regression model revealed that pregnant women aged 20 to <30 years and those with previous history of unfavorable pregnancy outcome were more likely to follow toxoplasmosis preventive behavior. Toxoplasmosis-related knowledge showed that many women had identified the role of cats in disease transmission while failed to identify other risk factors including consumption of undercooked meats, unwashed fruits and vegetables, and contacting with soil. Predictors for pregnant women to be knowledgeable towards toxoplasmosis included those aged 30 to <40 years (OR=1.53), with ≥ secondary education (OR=1.96), had previous unfavorable pregnancy outcomes (OR=1.88) and investigated for toxoplasmosis (OR=2.08) as reveled by multivariate regression model.

**Conclusion:**

Pregnant women in Al Hasas, Saudi Arabia, are substantially vulnerable to toxoplasmosis infection as they are lacking the necessary preventive behavior. A sizable portion have no sufficient knowledge for primary prevention of congenital toxoplasmosis, health education at primary care is necessary to avert the potential toxoplasmosis related complications especially in the neonates.

## 1. Introduction

Toxoplasmosis is a disease caused by an obligate intracellular protozoan parasite Toxoplasma *gondii* (Dubey, 2010). It is estimated that about one third of the World's population is infected with toxoplasmosis (Pappas et al., 2009). High prevalence of infection has been reported among pregnant women and women of childbearing age from different parts of the world including the Middle East (Pappas et al., 2009). In Saudi Arabia, the prevalence of infection showed wide variations as reveled from previous studies at different parts of the Kingdom. The highest positivity rate was reported in Jeddah 61.4% (Tonkal, 2008), Al Hassa of 51.4% (Al-Mohammed et al., 2010), and 35.6% in Makkah (Al-Harthi et al., 2006). Pregnant women with acute infection are at risk of congenitally transmitting the infection to the fetus (Lopez et al., 2000). Congenital toxoplasmosis is a rare condition, with low estimate but it can cause severe manifestations ranged from miscarriage to microcephaly, hydrocephalus, seizures, mental retardation and chorioretinitis (Cook et al., 2000). There is lack of information about the incidence of congenital toxoplasmosis in Saudi Arabia. Abdalla et al. (1995) found IgM positivity of 23.1% among the examined premature Saudi infants. In United Arab Emirates, Dar et al. (1997) have found an estimated prevalence of congenital toxoplasmosis of 12/1000 live births. In view of the asymptomatic nature of toxoplasmosis in adults and its high seroprevalence, primary prevention might decrease the likelihood of congenital toxoplasmosis (Baril et al., 1999). Several studies have addressed the contribution of various risk factors to toxoplasmosis seroconversion during pregnancy. These studies have found that the most significant risk factor to be undercooked meat consumption (Baril et al., 1999; Bobic et al., 1998; Bobic et al., 1996; Kravetz & Federman, 2005). One study estimated that up to 63% of the seroconversions during pregnancy was secondary to undercooked meat consumption (Jones et al., 2001), soil contact via gardening, eating raw or unwashed vegetables and fruits (Bobic et al., 1998; Bobic et al., 1996), contact with cat's litter and infrequent washing of kitchen knives used to cut raw meat (Bobic et al., 1998; Bobic et al., 1996; Kravetz & Federman, 2005). Centers for Diseases Control and Prevention (CDC) recommended the adoption of individual measures for primary prevention of toxoplasmosis during pregnancy, based the preventive behavior directed to food hygiene (Lopez et al., 2000). Nearly all studies that tackled toxoplasmosis among Saudi pregnant women estimated the prevalence of infection and its determinant factors (Tonkal, 2008; Al-Mohammed et al., 2010; Al-Harthi et al., 2006), while none have reported toxoplasmosis preventive behavior. Furthermore, studies assessed the state of toxoplasmosis-related knowledge among pregnant women in Saudi Arabia do not exist, such data are needed for initiating health education activities and aid in the control measures against congenital toxoplasmosis, especially in the absence of routine serological screening program. Dissemination of appropriate knowledge to prevent infection is necessary especially for those most vulnerable namely pregnant women. In a Belgian study it was found that health education was associated with a 63% reduction in toxoplasmosis seroconversion (Foulon et al., 1994). The objectives of this study were to assess the risk behavior and knowledge related to toxoplasmoisis among Saudi pregnant women attending primary health care centers (PHCs) in Al Hassa, Saudi Arabia and to determine socio-demographic characteristics related to risk behavior and knowledge.

## 2. Methods

### 2.1 Setting

This exploratory descriptive study was carried out in Al Hassa Governorate, Eastern Province of Saudi Arabia; 450 km from capital Riyadh, and populated by about 1.5 million, composed of three regions; urban, populated by about 60% of the total population, rural (23 villages with 35% of the population) and “Hegar” Bedouin scattered communities making up the remaining 5%.

### 2.2 Sampling

Epi-Info version 2002 (CDC, Atlanta, GA, U.S.A.) was used to calculate the sample size required. Pilot study reveled that 27% of pregnant women attending a nearby two PHCs (n=47) were adopting toxoplasmosis preventive behavior. Considering that the number of pregnant women registered for antenatal care in Al Hassa of 53778 (Local Health Directorate, 2011), with the worst acceptable level of preventive behavior of 23%, a minimal sample size of 500 were required, 20% were added for the possibility of none response, a total sample size of 610 pregnant were to be included, sampling fraction was employed for selection of subjects in relation to urban/rural distribution. Multistage sampling technique was employed, first, six urban and four rural PHCs were randomly chosen from an updated list (Hegar areas were excluded for transportation problems) and within each PHC all Saudi pregnant women attending for antenatal care from December 1st 2011 to May 31st 2012 were approached. Of the invited women (593 urban and 498 rural), 484 urban and 459 rural women agreed to participate.

### 2.3 Data Collection

Arabic speaking trained nurse at each PHC collected the data through a semi-structured interview including information regarding:

Socio-demographics: Age, residence, educational and occupational status, family income, maid in the house, number of living children, history of pregnancy wastage (abortions and intrauterine fetal deaths, IUFDs), number of pregnancies, and duration of the current pregnancy.

The second part of the interview included inquires about toxoplasmosis preventive behavior as recommended by Centers for Disease Control and Prevention and others (Lopez et al., 2000; Kravetz et al., 2005; Jones et al., 2001) for preventing congenital infection: Consumption of undercooked/partially cooked meat and its frequency in the last week before the interview, consumption of unwashed (and/or unpeeled) fruits and vegetables and their frequency in the last week, contacting with soil in the form of gardening (and whether with/without gloves), hand washing after gardening and after removal of gloves, sources of drinking water (bottled, wells or others), presence of cat in the house and their number, contacting with stray cats (if any), cleaning of domestic cat's litter, cleaning of kitchen utensils and cutting boards with hot soapy water. The question formats included yes/no and open ended questions.

Finally participants were inquired about toxoplasmosis-related knowledge employing items from the available literature (Kravetz et al., 2005; Jones et al., 2001; Jones et al., 2003) to assess their knowledge regarding epidemiology of the disease, its effect on pregnancy and the preventive measures to avert infection. The provisional questionnaire included four domains with 28 items included general information about the causative agent (4 items), risk factors (5), symptoms and signs (6), congenital toxoplasmosis (6), and preventive measures (7). Closed ended questions were used with true, false and don’t know or multiple options formats. For the knowledge section, correct answer assigned one point while incorrect or do not know responses assigned nil. At the end of the interview participants were inquired about their sources of information.

Revision of medical records to assess if women were ever being investigated, diagnosed or treated for toxoplasmosis, verifying the presence and nature of medical comorbidities (if any) and the occurrence of previous abortions and or IUFDs.

### 2.4 Pilot Testing

Initial questionnaire was compiled in English, which was translated into Arabic by two experts, and then back translated into English by another two independent experts to assure validity of constructs to be assessed. This form was tested on pregnant women attending two nearby PHCs (n=47) beyond the sample size to assure appropriateness, reliability, comprehensiveness and understanding by the interviewees. From the pilot testing toxoplasmosis is known among Saudi women as “cats’ disease”, the previous term was used all through the interview. Items from the initial knowledge-related questions were eliminated following the pilot testing including questions about signs and symptoms, nature of the organism (being protozoa), and time of maximum fetal damage if infection occurs during pregnancy. Elimination of items was done in response to “either total lack of knowledge and/or difficulties in understanding questions by the respondents”. Furthermore, based on alpha analysis which also showed low reliability coefficient; deletion of these items increase the internal consistency (Cronback's alpha) from.341 to.613. The final form consisted of 20 items: transmission (8 items), congenital infection (7items), and preventive measures (5 items).

### 2.5 Data Analysis

Total forms eligible for final analysis were 872, those with missing of ≥ two items were discarded (n=71). Data analysis was carried out using SPSS 16.0 (SPSS Inc, Chicago, IL, U.S.A.). For categorical data, frequency, proportions and percentage were used for reporting, Chi square test were used for comparison. For continuous variables, mean and standard deviation were reported and t-test was used for comparison. Pregnant women were categorized as having toxoplasmosis preventive behavior if they were found to fulfill all preventive behaviors recommended by the Centers for Diseases Control and Prevention 2000 to prevent congenital toxoplasmosis. Univariate analysis with reporting of Odds ratio (OR) and 95% confidence intervals (CI) was used to assess the possible associations of the included socio-demographic and obstetric-related factors with the adoption of toxoplasmosis preventive behavior. Multivariate logistic regression analysis model was generated to detect possible predictors for toxoplasmosis preventive behavior among the included pregnant women (dependent variable) in relation to the included socio-demographic and obstetric variables (independent variables) that were significant at univariate analysis. For the knowledge part, scores were expressed using median, mean and standard deviation and interquartile range (IQR), student, Mann Whitney and Kruslall Wallis tests were used for comparison when indicated. The 75^th^ percentile for the knowledge score was 8 points (out of 20) pregnant women attained a score of ≥ 8 points were considered as knowledgeable. A second multivariate regression model was generated to determine the possible correlates including socio-demographics, obstetric variables (independent variables) that predict the knowledge state (dependent variable). P value of < 0.05 was used to indicate statistical significance.

### 2.6 Ethical Considerations

Full orientation of the approached pregnant women about the study purposes was carried out with the emphasis on their right of not to participate. Informed consent forms were obtained and data confidentiality was maintained all though.

## 3. Results

### 3.1 Socio-Demographics and Obstetric Variables

The age of the included Saudi pregnant women (n=872) ranged from 17 to 48 years, mean of 29.7±8.4 years, 51.9% of them were urban, with illiteracy rate of 11.9%, educational status was higher among urban compared to rural women (P=0.001), 23.3% were in paid employments, family income was significantly higher in urban compared to rural women (P=0.002) and 26.5% were primigravide. Parity ranged from 0 to 12 with a median of 4.0, number of pregnancy with a median of 4.0 (4.3±3.1 for urban and 4.1±3.0 for rural women). Age at first pregnancy showed a median of 21.0 years (mean of 21.9±4.4 years), higher among rural women (21.6±3.8 years in urban vs. 22.2±5.1 years in rural). Of the interviewed pregnant women, 45.8% were in their 1^st^, 40.0% in their 2^nd^ and 16.2% in their 3^rd^ trimester of pregnancy. Encountered comorbid conditions included hypertension (6.0%), diabetes mellitus (5.5%), bronchial asthma (6.2%), and hemolytic blood diseases including sickle cell disease and thalassemia (more among rural women), 23.6% reported the presence of maids at houses (106 urban and 110 rural), 68% reported no previous bad pregnancy outcome (rural was higher the urban), while 32.0% mentioned the occurrence of previous bad pregnancy outcomes in the form of abortion and/or IUFD, ranged from 1 to 7. Of the included women 14.2% were investigated for toxoplasmosis during their previous/current pregnancies (significantly more among rural women), 31 women (4.2%) were being diagnosed as having toxoplasmosis during their previous pregnancies and 3.6% (31) had received treatment for infection (Table 1).

**Table 1:** Socio-demographics of the included pregnant women by residence, Al Hassa, Saudi Arabia

Characteristics	Total (N=872)	Residence: No. (%)		P value
		Rural (N=419)	Urban (N=453)	
**Age (years):** mean (SD):	29.7(8.4)	29.4(8.9)	29.9(8.5)	0.387^†^
**Age groups:**				0.806^!^
<20 years	79(9.1)	44(9.7)	35(8.4)	
≥20 & <30	165(39.4)	184(40.6)	349(40.0)	
≥30 & <40	148(35.3)	148(32.7)	296(33.9)	
≥40	71(16.9)	77(17.0)	148(17.0)	
**Educational status:**				0.001
Illiterate-Read and write	65(15.5)	39(8.6)	104(11.9)	
< Secondary	75(17.9)	45(9.9)	120(13.8)	
Secondary	156(37.2)	212(46.8)	368(42.2)	
College or higher	123(29.4)	157(34.6)	280(32.1)	
**Family income in Saudi Riyals:**				0.002
<3000	97(23.2)	52(11.4)	149(17.1)	
≥3000 & <6000	126(30.1)	130(28.7)	256(29.4)	
≥6000 & <10000	112(26.7)	154(34.0)	266(30.5)	
≥10000	84(20.0)	117(25.8)	201(23.1)	
**Working status:**				0.089
Housewife	284(67.8)	287(63.4)	571(65.5)	
Students	37(8.8)	61(13.5)	98(11.2)	
In Paid employment	98(23.4)	105(23.2)	203(23.3)	
**Parity**				0.065
1−3	184(43.9)	214(47.2)	398(45.6)	
>3 & <8	195(46.5)	174(38.4)	369(42.4)	
≥ 8	44(10.5)	61(13.4)	105(12.0)	
**Duration of pregnancy:**				0.251
1^st^ trimester	188(44.9)	211(46.6)	399(45.8)	
2^nd^ trimester	178(42.5)	171(37.7)	349(40.0)	
3^rd^ trimester	53(12.6)	71(15.7)	124(16.2)	
**Gravidity:**				0.011
Primigravidae	126(30.1)	105(23.2)	231(26.5)	
Multigravidae	239(69.9)	348(76.8)	641(73.5)	
Comorbidties *:				
Hypertension	23(5.5)	29(6.4)	52(6.0)	
Diabetes mellitus	21(5.0)	27(6.0)	48(5.5)	
Bronchial asthma	25(6.0)	29(6.4)	54(6.2)	
Hemolytic blood diseases (sickle-thalassemia)	26(6.2)	8(1.8)	34(3.9)	
More than one comorbidities	18(4.3)	19(4.2)	37(4.2)	
Others ^‡^	28(6.7)	43(9.5)	71(8.1)	
**Previous unfavorable obstetric outcomes:**				0.160
None	298(71.1)	295(65.1)	593(68.0)	
One abortion or intra-uterine foetal deaths (IUFD)	88(21.0)	117(25.8)	205(23.5)	
> one abortion or IUFD	33(7.9)	41(9.1)	74(8.5)	
Investigated for toxoplasmosis *	74(17.7)	50(11.0)	124(14.2)	0.006
Diagnosed as having toxoplasmosis *	19(4.5)	18(4.0)	37(4.2)	0.808
Received treatment for toxoplasmosis *	16(3.8)	15(3.3)	31(3.6)	0.824

SD=standard deviation,

! Chi-square,

† t-test.

* Based on reviewing of patients’ records.

‡ Includes nutritional anemia (32 cases), undernutrition (18), epilepsy (2), valvular heart disease (2 cases), joints problems (5 cases), and urinary and other infections (12 cases).

### 3.2 Toxoplasmosis Risk Behavior

Table 2 demonstrates toxoplasmosis risk behaviors by residence and duration of pregnancy. Of the included pregnant women 32.0% mentioned consumption of undercooked/partially cooked meat including Salami, Pastrami, luncheon, sausages, burgers, minced meats and similar foods, of them 17.4% stated a frequency consumption of at least once/week (median of 2.5 times/week), this was more among rural women and those in their 3^rd^ trimester but without statistical significance. Also, 44.2% stated eating of unwashed/unpeeled fruits and vegetables, with consumption of at least once/week in 28.2% significantly more among rural women, and those women in the 2^nd^ and 3^rd^ trimesters of pregnancy. Of the included women 11.3% mentioned using water wells for drinking significantly higher in rural women. Only 3.9% of pregnant women owned domestic cats, more in urban and only 16 (1.8%) of the interviewed women mentioned changing the cat's litters. Gardening without gloves was stated by 9.1% of the women significantly more among rural (P=0.001) and those 1^st^ and 2^nd^ trimesters compared to those in their 3^rd^ trimester of pregnancy. An additional 6.7% mentioned using gloves while gardening but without washing their hands. Cleaning of kitchen utensils using hot soapy water was mentioned by 57.0%, more among urban women, least among women in their 1^st^ trimester of pregnancy. Thorough washing of fruits and vegetables before eating was mentioned by 64.6%, more among urban and least among those of 1^st^ trimester but without statistical significance. Washing hands after contact with raw meats/unwashed fruits and vegetables was mentioned by 65.6% and least among those in their 3^rd^ trimester.

**Table 2:** Toxoplasmosis risk behaviors among Saudi pregnant women by residence and duration of pregnancy, Al Hassa, Saudi Arabia

Behaviors	Total (N=872)		Residence: No. (%)		P value*	Trimester of pregnancy: No.(%)			P value*
	
	No. (%)	95% CI	Urban (N=453)	Rural (N=419)		1^st^ (N=399)	2^nd^ (N=349)	3^rd^ (N=124)	
**Eating under/partially cooked meat:**									
At least once a week	152(17.4)	15.1-20.1	71(15.7)	81(19.3)	0.154	63(15.8)	62(17.8)	27(21.8)	0.301
Less frequent (1-3 times/month).	127(14.6)	12.4-17.1	78(17.2)	49(11.7)	0.020	55(13.8)	49(14.0)	23(18.5)	0.395
**Eating unwashed raw/non peeled fruits and vegetables:**									
At least once a week	246(28.2)	25.3-31.3	102(22.5)	144(34.4)	0.001	114(28.6)	101(28.9)	31(25.0)	0.687
Less frequent (1-3 times/month).	139(15.9)	13.7-18.5	72(15.9)	67(16.0)	0.969	74(18.5)	33(9.5)	32(25.8)	0.001
**Drinking water sources:**									
Water wells (private or public):	99(11.3)	9.4-13.6	12(2.6)	87(20.8)	0.001	47(11.8)	41(11.7)	11(8.9)	0.642
Others (mobile or bottled water):	773(88.7)	86.4-90.6	441(97.4)	332(79.2)	0.001	352(88.2)	278(79.7)	113(91.1)	0.474
**Cat ownership**	34(3.9)	2.8-5.4	21(4.6)	13(3.1)	0.245	17(4.3)	16(4.6)	1(0.8)	0.153
Changing cat's litter	16(1.8)	1.1-3.0	7(1.5)	9(2.1)	0.507	9(2.3)	7(2.0)	-	0.813
**Contacting with soil:**									
Garden at the house	193(22.1)	19.5-25.0	89(19.6)	104(24.8)	0.065	88(22.1)	71(20.3)	34(27.4)	0.264
Gardening without gloves	79(9.1)	7.3-11.1	25(5.5)	54(12.9)	0.001	31(7.8)	30(8.6)	8(6.5)	0.741
Gardening with gloves but without hand washing	58(6.7)	5.2-8.5	25(5.5)	33(7.9)	0.162	28(7.3)	23(6.6)	6(4.8)	0.691
Cleaning kitchen utensils (knives/cutting boards/utensils/counters):	497(57.0)	53.7-60.2	268(59.2)	229(54.7)	0.179	198(49.6)	237(67.9)	62(50.0)	0.001
Thorough washing of fruits and vegetables before eating:	563(64.6)	61.3-67.7	301(66.4)	262(62.5)	0.227	241(60.4)	239(68.5)	83(66.9)	0.058
Washing hands after contact with raw meats/unwashed fruits and vegetables:	574(65.8)	62.6-68.9	296(65.3)	278(66.3)	0.754	262(65.7)	241(69.1)	71(57.3)	0.058

The term cats’ illness (disease) was used to indicate toxoplasmosis. CI = confidence intervals

* Chi Square test.

### 3.3 Correlates of Toxoplasmosis Preventive Behavior

Saudi pregnant women fulfilled the recommended behaviors to prevent congenital toxoplasmosis constituted 26.8% (n=234), significantly more among urban compared to rural women (30.0% vs. 23.4% rural) but not associated with the duration of pregnancy (97/399, 24.3% among those in the 1^st^ trimester, 101/349, 28.9% in the 2^nd^ and 36/124, 29.0% in the 3^rd^, P=0.304). Of the included women, 426(48.9%) had at least one risk behavior, while those with ≥ two risk behaviors constituted 24.3% (n=212), more among rural women (28.4% compared to 20.6% urban, P=0.010). The preventive behavior is largely stemmed from not contacting with cat and cat's litter (98.2%), not contacting with soil (90.9%), safe water supply (88.7%), not eating undercooked and raw meat (82.6%), and no consumption of unwashed/unpeeled fruits and vegetables (71.8%).

Table 3 displays univariate and multivariate regression model of the possible correlates for toxoplasmosis preventive behavior. Pregnant urban women aged 20 to <30 years (Odds ratio OR=2.37, confidence intervals CI=1.72-3.28), with ≥ secondary education (OR=1.64, CI=1.12-2.41) and with history of two or more abortion and/or IUFDs (OR=5.33, CI=3.16-9.03) were more likely to follow preventive behavior as revealed by univariate analysis. Multivariate logistic regression model demonstrates that pregnant women with ≥ secondary education (OR=1.59), aged 20 to < 30 years (OR=2.11) and those with two or more abortions and/or IUFDs (OR=4.7) were positive predictors for adopting toxoplasmosis preventive behavior.

**Table 3:** Possible socio-demographic and obstetric predictors for toxoplasmosis preventive among the included Saudi pregnant women, Al Hassa, Saudi Arabia

Variables	Preventive behavior^!^: No. (%)		Univaraite analysis Odds ratio (95% CI)	Multivariate logistic regression Odds ratio (95% CI)
	Yes (N=234)	No (N=638)		
Age groups:						
<20 years	30(12.8)	49(7.7)	Reference	Reference
≥20 & <30	137(58.5)	212(33.2)	2.37(1.72-3.28)**	2.11(1.63-2.74)**
≥ 30	66(28.2)	306(48.0)	0.34(0.24-0.58)**	0.33(0.21-0.51)**
**Residence:**				
Rural	98(41.9)	321(50.3)	Reference	Reference
Urban	136(58.1)	317(49.7)	1.41(1.03-1.92)*	1.37(0.96-1.90)
**Educational status:**				
< Secondary	45(19.2)	179(28.1)	Reference	Reference
Secondary or higher	189(80.8)	459(71.9)	1.64(1.12-2.41)*	1.59(1.08-2.35)*
**Family income in Saudi Riyals**				
<3000	31(13.2)	118(18.5)	Reference	--
≥3000 & <6000	66(28.2)	190(29.8)	0.93(0.66-1.31)	--
≥6000	137(58.5)	330(51.7)	1.32(0.96-1.89)	--
**Working status:**				
Housewife/Students	176(75.2)	493(77.3)	Reference	--
In Paid employment	58(24.8)	145(22.7)	1.12(0.78-1.61)	--
**Parity**				
1-3	111(47.4)	287(45.0)	Reference	--
≥4 & <8	92(39.4)	277(43.4)	0.84(0.61-1.16)	--
≥ 8	31(13.2)	74(11.6)	1.16(0.72-1.86)	--
**Previous unfavorable obstetric outcomes:**				
None	172(73.5)	421(66.0)	Reference	Reference
One abortion or IUFD	16(6.8)	189(29.6)	0.17(0.10-0.31)**	0.14(0.10-0.30)**
> One abortion or IUFD	46(19.7)	28(4.4)	5.33(3.16-9.03)**	4.71(3.09-7.19)**
**Previously investigated for toxoplasmosis:**	28(12.0)	96(15.0)	0.77(0.48-1.23)	--

CI= Confidence intervals

* P < 0.05,

** P< 0.001.

! Fulfilling all criteria recommended by the Centers for Disease Control and Prevention to prevent congenital toxoplasmosis.

### 3.4 Toxoplasmosis-Related Knowledge

Table 4 displays the correct responses regarding toxoplasmosis-related knowledge stated by pregnant women and distributed by their residence and duration of pregnancy. The majority of the pregnant women failed to identify the role of undercooked/partially cooked meat consumption, contaminated water, eating unwashed fruits and vegetables and contacting with soil in transmitting infection. Only 13.4% correctly stated that toxoplasmosis can produce serious complications in the unborn child, 10.2% recognized that toxoplasmosis can pass to the fetus if the pregnant women is newly infected. The domain of knowledge related to toxoplasmosis prevention showed higher correct responses compared to infection transmission and congenital toxoplasmosis. The total knowledge score ranged from 0 to 16 (out of 20 points), median of 5.0, IQR=7.0, mean (SD) 4.9±2.7, higher among urban (mean 5.1±2.8) compared to rural (4.6±1.7) (Mann Whitney, P=0.006). The knowledge score was significantly correlated with the duration of pregnancy, least among those in the 1^st^ trimester compared to those in the 2^nd^ and 3^rd^ trimester (Kruskal Wallis, P=0.015).

**Table 4:** Toxoplasmosis related knowledge among pregnant women by residence and duration of pregnancy, Al Hassa, Saudi Arabia

Items	Correct responses: No. (%)			P value*	Trimester of pregnancy: No. (%)			P value*
	
	Total (N=872)	Urban (N=453)	Rural (N=419)		1^st^ (N=399)	2^nd^ (N=349)	3^rd^ (N=124)	
**Ever heard/read about toxoplasmosis:**	367(42.1)	178(39.3)	185(44.2)	0.165	161(40.3)	149(42.7)	57(46.0)	0.518
**Infection transmission:**								
Toxoplasmosis is caused by___ (options): **Infection**	148(17.0)	88(19.4)	60(14.3)	0.055	61(15.2)	60(17.2)	27(21.8)	0.241
Dealing with cats increase the risk of infection: **True**	341(39.1)	182(40.2)	159(37.9)	0.545	159(39.8)	146(40.1)	42(33.9)	0.297
Toxoplasmosis agent is present in cats’ feaces: **True**	304(34.9)	166(36.6)	138(32.9)	0.281	132(33.1)	123(35.2)	49(39.5)	0.414
Toxoplasmosis agent can be found in undercooked meat: **True**	133(15.3)	70(15.5)	63(15.0)	0.938	60(15.0)	53(15.2)	20(16.1)	0.956
Eating undercooked meat increases the risk of toxoplasmosis: **True**	129(14.8)	61(13.5)	46(11.0)	0.310	53(13.3)	53(15.2)	23(18.5)	0.341
Drinking water contaminated with toxoplasmosis increases the risk: **True**	83(9.5)	37(8.2)	46(11.0)	0.194	31(7.8)	33(9.5)	19(15.3)	0.043
Eating unwashed fruits and vegetables increases risk of toxoplasmosis: **True**	66(7.6)	39(8.6)	27(6.4)	0.280	28(7.0)	26(7.4)	13(10.5)	0.438
Gardening without gloves may increases risk of toxoplasmosis: **True**	101(11.6)	59(13.0)	42(10.0)	0.201	41(10.3)	47(13.5)	13(10.5)	0.363
**Congenital toxoplasmosis:**								
Toxoplasmosis can cause severe symptoms to the pregnant: **False**	147(16.9)	92(20.3)	55(13.1)	0.006	54(13.5)	67(19.2)	26(21.0)	0.049
Toxoplasmosis can produce serious complications in unborn /newborn child: **True**	117(13.4)	71(15.7)	46(11.0)	0.053	47(11.8)	49(14.0)	21(16.9)	0.307
Toxoplasmosis can pass to the fetus if the pregnant is newly infected. **True**	89(10.2)	44(9.7)	45(10.7)	0.697	32(8.0)	39(11.2)	18(14.5)	0.084
Chronically infected pregnant women rarely pass toxoplasmosis to her fetus. **True**	83(9.5)	51(11.2)	32(7.6)	0.088	28(7.0)	38(10.9)	17(13.7)	0.045
A baby with Toxoplasmosis may have no signs of illness at birth, but develop latter. **True**	121(13.9)	68(15.0)	53(12.6)	0.362	45(11.3)	49(14.0)	27(21.8)	0.012
A baby with toxoplasmosis can have visual problems: **True**	102(11.7)	50(11.0)	52(12.4)	0.599	33(8.3)	41(11.7)	28(22.6)	0.008
A baby with toxoplasmosis can have mental problems: **True**	119(13.6)	60(13.2)	59(14.1)	0.794	43(10.8)	52(14.9)	24(19.4)	0.035
**Toxoplasmosis prevention:**								
Toxoplasmosis can be prevented by avoid dealing with cat's feaces: **True**	289(33.1)	186(41.1)	103(24.6)	0.111	103(25.8)	133(38.1)	53(42.7)	0.001
Thorough cooking of meat until no pink is seen can prevent toxoplasmosis: **True**	231(26.5)	133(29.4)	98(23.4)	0.054	101(25.3)	96(27.5)	34(27.4)	0.769
Avoid eating of raw/undercooked meat can prevent toxoplasmosis: **True**	181(20.8)	94(20.8)	87(20.8)	0.937	79(19.8)	71(20.3)	31(25.0)	0.445
Thorough washing and /or peeling of fruits and vegetables before eating can prevent toxoplasmosis: **True**	277(31.7)	159(35.1)	118(28.2)	0.033	123(30.8)	106(30.4)	48(38.7)	0.198
Cleaning of knives, cutting boards and utensils after each use can prevent toxoplasmosis. **True**	267(30.6)	148(32.7)	119(28.4)	0.195	142(35.6)	102(29.2)	41(33.1)	0.179
Knowledge score: median (mean ± SD) Interquartile range (25^th^ -75^th^ percentiles)	5.0(4.9±2.7) 1.0-8.0	5.0(5.1±2.8) 1.0-8.0	4.0(4.6±1.7) 0.0-7.0	0.006^!^	4.0(4.3±1.4) 0.0-7.0	5.0(4.8±2.1) 1.0-8.0	5.25(4.9±2.8) 1.0-8.0	0.015^†^

* Chi-square test.

! Mann Whitney,

† Kruskal Wallis test.

Table 5 demonstrates the univaraite analysis of socio-demographics and obstetric factors against the knowledge scores applying cut of ≥ 8 points for being knowledgeable to toxoplasmosis. Of the included pregnant women, 219 (25.1%) scored 8 or more points for knowledge. Univaraite analysis showed that urban woman, within the age range of 30-<40 years, with ≥ secondary education, having previous unfavorable pregnancy outcomes, and being previously investigated for toxoplasmosis were more knowledgeable. Those in the 1^st^ trimester of pregnancy were the least likely to be knowledgeable (OR=0.39, CI=0.28-0.55, P=0.001). Family income, parity, working status and preventive behavior were not significantly correlated with the knowledge status. Also table 5 displays the results of multivariate logistic regression model showed that women aged 30 to <40 years (OR=1.53, CI=1.09-2.15), with ≥ secondary education (OR=1.96, CI=1.54-2.49), previous history of unfavorable pregnancy outcomes (OR=1.88, CI=1.13-2.99) and previously investigated for toxoplasmosis (OR=2.08, CI=1.61-2.67) were significantly more knowledgeable towards toxoplasmosis.

**Table 5:** Univariate and multivariate logistic regression analysis of the possible socio-demographic and obstetric history correlates of toxoplasmosis-related knowledge among the included pregnant women, Al Hassa, Saudi Arabia

Independent variables	Knowledge level: No. (%)		Univariate analysis (Odds ratio and 95% CI)	P value	Multivariate regression model Odds (95% CI)	P value

	Low (<8) (N=653)	High (≥8) (N=219)				
Residence:						
Rural	336(51.5)	83(37.9)	Reference		Reference	
Urban	317(48.5)	136(62.1)	1.74(1.25-2.41)	0.001	1.55(0.89-2.36)	0.115
**Age groups:**						
<20	68(10.4)	11(5.0)	Reference		Reference	
≥20 & <30	265(40.6)	84(38.4)	0.91(0.66-1.26)	0.090	0.87(0.61-1.24)	0.360
≥30 & <40	204(31.2)	92(42.0)	1.59(1.15-2.21)	0.003	1.53(1.09-2.15)	0.034
≥ 40	116(17.8)	32(14.6)	0.79(0.51-1.24)	0.282	0.73(0.48-1.12)	0.287
**Educational status:**						
< secondary	194(29.7)	30(13.7)	Reference		Reference	0.006
Secondary or higher	459(70.3)	189(86.3)	2.66(1.72-4.15)	0.001	1.96(1.54-2.49)	
**Family income:**						
<6000 SR	299(45.8)	106(48.4)	Reference			
≥6000 SR	354(54.2)	113(51.6)	0.91(0.66-1.25)	0.538	--	
**Working:**						
Housewives/students	508(77.8)	161(73.5)	Reference			
Working	145(22.2)	58(26.5)	1.26(0.91-1.72)	0.190	--	
**Parity:**						
1-3	289(44.3)	109(49.8)	Reference			
4 or more	364(55.7)	110(50.2)	0.80(0.58-1.10)	0.156	--	
**Trimester of pregnancy:**						
1st	235(51.3)	64(29.2)	Reference		Reference	
2nd	232(35.5)	117(53.4)	1.58(1.14-2.19)	0.002	1.43(0.98-2.09)	0.091
3rd	86(13.2)	38(17.4)	1.14(0.73-1.70)	0.539	1.07(0.69-1.66)	0.601
**Previous unfavorable pregnancy outcomes:**						
None	480(73.5)	113(51.6)	Reference		Reference	
One or more	173(26.5)	106(48.4)	2.60(1.87-3.62)	0.001	1.88(1.13-2.99)	0.016
**Previously investigated for toxoplasmosis during pregnancy:**						
None	582(89.1)	166(75.8)	Reference		Reference	
Yes	71(10.9)	53(24.2)	2.62(1.73-3.96)	0.001	2.08(1.61-2.67)	0.009
**Preventive behavior:**						
No	480(73.5)	158(72.1)	Reference			
Yes	173(26.5)	61(27.9)	1.07(0.75-1.53)	0.694	--	

CI=Confidence intervals. Percent predicted for the multivariate regression model=75.8, Homser and Lemeshow Chi square=4.539, P=0.602

**Figure 1:**
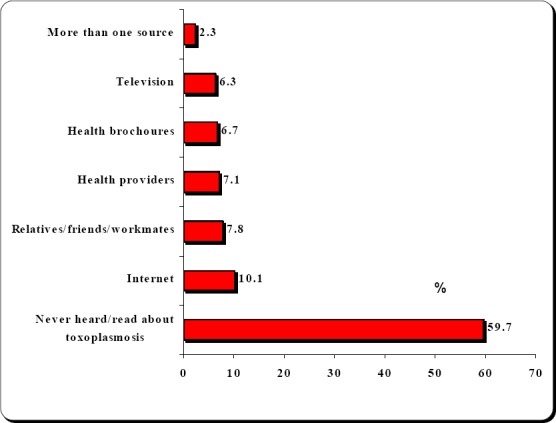
Sources of information regarding toxoplasmosis cited by the included Saudi pregnant women, Al Hassa, Saudi Arabia

Figure 1 displays the cited sources of toxoplasmosis-related knowledge cited by the included Saudi pregnant women, 521 (59.7%) stated that they never heard/read about toxoplasmosis, while 351 (40.3%) had mentioned that they have read/hear about the condition and cited their sources of toxoplasmosis-related knowledge. These sources included Television programs 55/351 (15.7%), Internet by 88/351 (25.1), close friends/family relatives and workmate 68/351 (19.4%), health related booklets, brochures and papers 58/351 (16.5%), health care providers by 62/351 (17.7%) and 20/351 (5.6%) had cited more than one source.

## 4. Discussion

This study shows that toxoplasmosis risk behavior among the included Saudi pregnant women is largely stemmed form hygienic habits in the form of non-cleaning of kitchen utensils, non washing of hands after dealing with raw meats and vegetables, and non thorough washing of fruits and vegetables. Studies from Turkey (Ertug et al., 2005) and Palestine (Nijem & Al-Amleh, 2009) reported a positive association between raw meats consumption with toxoplasmosis infection. Al Qahtani and Hassan (2012) explained the urban-rural difference in toxoplasmosis seroprevalence by the differential distribution of risk factors in Nigran (Southern Saudi Arabia) including frequent consumption of undercooked meat, unpasteurized milk, unfiltered municipal water, raw or non-washed vegetables or fruit and soil contact. Moreover, higher number of cats in rural areas especially inside houses may be responsible for that higher difference in rural localities (Al-Harthi et al., 2006). Understanding the preventive behavior of pregnant women may aid in counseling which of paramount importance in lowering the fetal risk. In this study the proportion of pregnant women followed a preventive behavior is higher compared to those reported from Brazil 16.3% of pregnant women demonstrated preventive behavior (Costa et al., 2012). In this study pregnant women aged 20 to <30 years and those with repeated abortions or intrauterine fetal deaths were more likely to follow preventive behavior. These findings are consistent with those reported from one study in other Brazil (Costa et al., 2012) where no significant associations were found between preventive behavior with sociodemographics, gestational age and IgG positivity. Other study had linked preventive behavior for toxoplasmosis with eating and hygienic habits (Carneil et al., 2006). Belarmino et al. (2009) have reported that preventive behavior for toxoplasmosis among pregnant women was chiefly stemmed from the changes in the eating habits that occur during pregnancy, while others postulated that non-preventive behavior among pregnant women resulted from the lack of information (Costa et al., 2012; Belarmino et al., 2009). Low family income and little formal schooling among the pregnant women can contribute to increase the risk for infection (Costa et al., 2012). Our results showed that ≥ secondary education was significantly associated with higher preventive behavior, while family income did not. Toxoplasmosis was considered by many gynecologists in the developing countries as the primary cause of bad obstetric outcome; this is viewed by the public and had created a type of panic reaction among population and especially women (Razzak et al., 2005). Women with bad obstetric history attributed abortion and IUFDs to toxoplasmosis and hence seek care for investigating and even treatment of toxoplasmosis without physician referral (Giofangrandi et al., 1994). The previous notion may partially explain the effect of bad obstetric history in the form of repeated abortions and/or IUFDs on both preventive behavior and the state of knowledge as reveled from this study.

In this study pregnant women cited that contacting with cat and changing cat's litter to be the most likely method for transmission while most of them failed to identify the roles of eating undercooked meat, soil contacting and consumption of unwashed fruits and vegetables. The results of the current study are close to those reported from Brazil where 65% of included pregnant women did not know about the disease and most of them associated toxoplasmosis with having cats as pets and less than one third of the participants associated the disease with handling and consumption of undercooked meats and raw vegetables (Varella et al., 2009). Also, Jones et al in their survey showed that 60% of pregnant women associated transmission of toxoplasmosis to cats and only 30% were aware of the risk of acquiring the infection from raw or undercooked meat (Jones et al., 2001). Adequate knowledge of risk factors for toxoplasmosis infection supports the preventive measures against severe complications resulted from congenital infection and only with this knowledge can pregnant women lower the risk for fetal infection (Kravtz et al., 2005).

In this study the highest level of knowledge was about the association between cats and toxoplasmosis with low level of knowledge about the role of other modes of transmission. Similar results were reported among U.S pregnant women (Jones et al., 2001) and even among obstetricians and gynecologists (Jones et al., 2003). Also this study demonstrated that many women have shown fair knowledge regarding the preventive practices which is consistent with a U.S study where the majority of the included women picked up correct preventive practices to avoid infection. This study as well other confirmed that disease specific knowledge was not necessarily associated with preventive behavior during pregnancy, regarding toxoplasmosis and conversely, a lack of knowledge was not always associated with engaging in risk behavior. Health education for infectious diseases especially those with grave complications are to be considered early in the antenatal period (Kravtz et al., 2005; Jones et al., 2001; Jones et al., 2003). A Canadian study has shown that even brief education of pregnant women helps to improve toxoplasmosis preventive behaviors (Carter et al., 1989). Health education was associated with reduction in toxoplasmosis seroconversion (Foulon et al., 1994), hence the importance of providing health education as a tool in prevention of congenital toxoplasmosis. Education about meat-cat-soil-related hygiene should be provided to women of childbearing age and to pregnant women at their first prenatal visit. It is important that the educational materials be complete and accurate and that they be made available in a culturally and linguistic appropriate format (Foulon et al., 1994; Pawlowski et al., 2001).

## 5. Study Limitations

To the authors’ knowledge this is the first primary care-based study that explores toxoplasmosis-related preventive behavior and knowledge among pregnant women in Saudi Arabia. Nevertheless, the results of the current study should be cautiously interpreted in the lights of the following limitations: First, the study population was not representative of all pregnant women in Saudi Arabia. Second, possible inherent limitations of the study design with the potential recall bias and over/under reporting of the included risk and preventive behavior. Third, the behavioral questions may have contributed to social desirability bias, which may have affected the frequency of the reported risk behavior among the included sample. Moreover, the questionnaire design especially with regard to knowledge about toxoplasmosis was largely based on true/false and do not know format options with high probability of guessing which we did not control, validation of the such instruments need further studies. Fourth, causal relationship between risk behavior, seroconversion and/or pregnancy outcome were not carried out which imply more cautious interpretation of the results. Finally, the effects of adopting risk and preventive behavior were not assessed due to nature of the study design.

## 6. Conclusion

Pregnant women in Al Hasas, Saudi Arabia, are substantially vulnerable to toxoplasmosis infection as they are lacking the necessary preventive behavior. A sizable portion have no sufficient knowledge for primary prevention of congenital toxoplasmosis, health education at primary care is necessary to avert the potential toxoplasmosis related complications especially in the neonates.
